# Compositional Study and Antioxidant Potential of *Ipomoea hederacea* Jacq. and *Lepidium sativum* L. Seeds

**DOI:** 10.3390/molecules170910306

**Published:** 2012-08-29

**Authors:** Muhammad Zia-Ul-Haq, Shakeel Ahmad, Luca Calani, Teresa Mazzeo, Daniele Del Rio, Nicoletta Pellegrini, Vincenzo De Feo

**Affiliations:** 1 Department of Pharmacognosy, Research Institute of Pharmaceutical Sciences, University of Karachi, Karachi-75270, Pakistan; Email: ahirzia@gmail.com; 2 Department of Agronomy, Bahauddin Zakariya University, Multan-60800, Pakistan; Email: shakeel.agronomy@gmail.com; 3 Department of Public Health, University of Parma, via Volturno 39, 43125 Parma, Italy; Email: luca.calani@nemo.unipr.it (L.C.); mazfer@libero.it (T.M.); daniele.delrio@unipr.it (D.D.R.); nicoletta.pellegrini@unipr.it (N.P.); 4 Department of Pharmaceutical and Biomedical Sciences, Salerno University, Fisciano, 84084 Salerno, Italy; Email: defeo@unisa.it

**Keywords:** *Ipomoea hederacea* Jacq., *Lepidium sativum* L., antioxidant capacity, proximate composition

## Abstract

The present investigation has been carried out to determine the proximate composition, amino acids, metal contents, oil composition as well as the antioxidant capacity of the seeds of *Ipomoea hederacea* Jacq. and *Lepidium sativum* L. Proximate composition indicated a great difference in oil (14.09 ± 0.66, 28.03 ± 1.05) and fibre (16.55 ± 0.31, 6.75 ± 1.20) contents for *I. hederacea* and *L. sativum*, respectively. Fatty acid profile indicated that oleic acid (19.50 ± 0.37, 30.50 ± 0.16) and linoleic acid (52.09 ± 0.48, 8.60 ± 0.38) are the major fatty acids. γ-Tocopherol and δ-tocopherol (28.70 ± 0.14, 111.56 ± 0.37) were the most abundant in the seed oil of *I. hederacea* and *L. sativum*, respectively. Results of TEAC, FRAP and TRAP antioxidant assays indicated that *L. sativum* has much greater antioxidant potential than *I. hederacea*.

## 1. Introduction

*Lepidium sativum* L. (*Brassicaceae*) is a fast growing annual herb that is native to Egypt and west Asia, although it is now cultivated in the entire World. Its young leaves are eaten raw or cooked, while its seeds are used, fresh or dried, as a seasoning with a peppery flavor [1]. Boiled seeds are consumed in drinks, either ground in honey or as an infusion in hot milk. Its root is used as a condiment while shoots are used in sandwiches [2,3]. The seed paste is applied to rheumatic joints to relieve pain and swelling. The seeds are chewed to treat sore throats, coughs, asthma and headaches, and in large quantities to induce abortion. Seeds pounded in water are used to treat hiccoughs and stomach-aches. The seeds are also considered galactagogue, emmenagogue and laxative and used a dressing for sores on camels and horses [4]. A further use is to prepare a solution to massage infected cows’ udders. The seed oil is used as an illuminant and in soap manufacture [5]. Recently, the seed structure of this plant has been reported [6], as well as the seed metabolism during germination [7].

*Ipomoea hederacea* Jacq. (*Convolvulaceae*) seeds have diuretic, anthelmintic, and blood purifier properties and are used for treating constipation, dropsy, to produce abortion and promote menstruation. They are laxative, carminative, cure inflammations, abdominal diseases, fevers, headaches and bronchitis. Juice of the leaves is also used for the same purpose. Seeds are useful in skin diseases like leucoderma and scabies, gout, cephalalgia, hepatopathy and splenopathy [8,9,10].

Antioxidants are an important part of the defense system of the human body and help to cope with oxidative stress caused by reactive oxygen species. Plants are important sources of antioxidants and there is increasing interest in antioxidant analysis of plants. Very little data on compositional studies and the antioxidant potential of these two species exists. As part of our studies to explore the composition and antioxidant potential of the indigenous flora of Pakistan [11,12,13,14,15,16,17,18], this research was carried out to study *I. hederacea* Jacq. and *L. sativum* L. seeds for nutritional composition and antioxidant capacity. 

## 2. Results and Discussion

The proximate composition of *I. hederacea* and *L. sativum* seeds (Table 1) indicates the presence of appreciable amounts of protein (23.36 ± 1.02% to 24.18 ± 1.54%), fibre (16.55 ± 0.31% to 6.75 ± 1.02%), lipids (14.09 ± 0.66% to 28.03 ± 1.05%), ash (3.65 ± 1.10% to 3.92 ± 1.06%), moisture (5.29 ± 1.02% to 3.92 ± 1.06%) and carbohydrates (37.06 ± 0.89% to 32.87 ± 0.29%). Proximate composition varies depending upon plant variety, agronomic practices, stage of collection of seeds and climatic and geological condition of area from where seeds are collected. It is an important indicator and first step during nutritional evaluation of fruits and seeds of plants and crops and it dictates further studies on components which seem more interesting [14,16].

Ash contents indicate the seeds are an appreciable source of minerals. The low moisture content is an index of stability, quality and increased shelf life of seeds [19]. Our results are in partial agreement with those reported earlier for *I. hederacea* [20,21] and *L. sativum* [22]. Higher protein and lipid contents indicate that both seeds have high food energy, however seeds cannot be considered safe for consumption since the toxicity levels for the seeds are not established yet.

**Table 1:** Proximate chemical composition of seeds (%).

Component	*Ipomoea hederacea*	*Lepidium sativum*
Crude protein	23.36 ± 1.02 b	24.18 ± 1.54 a
Total lipids	14.09 ± 0.66 b	28.03 ± 1.05 a
Total carbohydrates	37.06 ± 0.89 a	32.87 ± 0.29 b
Crude fiber	16.55 ± 0.31 a	6.75 ± 1.02 b
Moisture	5.29 ± 1.02 a	3.92 ±1.06 b
Ash	3.65 ± 1.10 a	4.25 ± 0.13 a

Data are expressed as the mean ± standard deviation; values in the same row having different letters differ significantly (*p* < 0.05).

The relatively higher contents of proteins and lipids motivated us to undertake in-depth studies on both types of biomolecules. Data concerning qualitative and quantitative amino acids composition is presented in Table 2. Amino acid composition indicates the nutritional quality of protein. Glutamic acid and aspartic acid were found to be the major non-essential amino acids in the samples tested. Results indicated that all essential amino acids, except S-containing types and tryptophan, are present in high amounts in both species. The amino acid profile showed that methionine and cysteine are present in the lowest concentration. Results are comparable to those of earlier workers for *L. sativum* [23,24] while there is no previous report on amino acid composition of *I. hederacea* seeds.

**Table 2:** Percentage composition of amino acids in seeds.

Amino acid	*Ipomoea hederacea*	*Lepidium sativum*
Isoleucine	5.03 ± 0.36 a	4.19 ± 0.03 b
Leucine	6.59 ± 0.85 b	7.03 ± 0.08 a
Lysine	4.25 ± 0.54 b	5.98 ± 0.03 a
Methionine	1.17 ± 0.62 a	0.51 ± 0.01 b
Phenylaniline	6.24 ± 0.92a	5.39 ± 0.17 b
Threonine	3.07 ± 0.31 a	3.76 ± 0.08 a
Tryptophan	1.88 ± 0.05 a	0.92 ± 0.07 b
Valine	7.10 ± 0.94 a	6.21 ± 0.02 b
Arginine	5.50 ± 0.23 a	3.44 ± 0.11 b
Histidine	3.55 ± 0.92 a	3.87 ± 0.14 a
Alanine	3.99 ± 0.37 b	4.59 ± 0.19 a
Aspartic acid	10.82 ± 0.86 b	12.07 ± 0.31 a
Cystine	0.90 ± 0.01 a	0.21 ± 0.11 a
Glutamic acid	22.71 ± 0.41 b	24.29 ± 0.75 a
Glycine	5. 36 ± 0.39 a	5.08 ± 0.12 a
Proline	4.46 ± 0.53 a	4.63 ± 0.36 a
Serine	4.02 ± 0.46 a	4.18 ± 0.55 a
Tyrosine	2.58 ± 0.59 a	2.88 ± 0.69 a

Data are expressed as the mean ± standard deviation; values in the same row having different letters differ significantly (*p* < 0.05).

Mineral contents of seeds (Table 3) varied between both species, but potassium constituted the major mineral in both cases, ranging from 978.46 mg/100 g in *I. hederacea* to 1,236.51 mg/100 g in *L. sativum*. Zinc was found in the lowest quantity in *I. hederacea* (4.0 mg/100 g), while *L. sativum* had the lowest manganese content (2.0 mg/100 g) content. Both species contained good amounts of calcium, phosphorus and magnesium. Mineral content for *L. sativum* is in agreement with those reported earlier for *L. sativum* [22], while the metal content of *I. hederacea* has not been reported previously. The relatively high amount of sodium in *I. hederacea* seeds indicates that its high consumption might cause problems for hypertensive patients. Considering the different elements present in seeds of both plants, both plants have potential for providing essential nutrients for human and other animals, since the nutritional activity of any plant is usually related to the particular elements it contains [25].

**Table 3:** Mineral content (mg/100 g) of seeds.

Metals	*Ipomoea hederacea*	*Lepidium sativum*
Calcium	317.41 ± 1.72 b	266.35 ± 1.44 a
Copper	4.62 ± 0.64 b	5.73 ± 2.11 a
Iron	9.85 ± 1.02 a	8.31 ± 0.36 a
Magnesium	179.14 ± 1.23 b	339.23 ± 2.13 a
Manganese	6.37 ± 0.68 a	2.00 ± 1.08 b
Phosphorus	596.19 ± 2.36 a	608.63 ± 1.39 a
Potassium	978.46 ± 1.44 b	1236.51 ± 1.67 a
Sodium	106.32 ± 1.33 a	19. 65 ± 0.98 b
Zinc	4.01 ± 0.69 b	6.99 ± 0.54 a
Na:K	0.11	0.01
Ca:P	0.53	0.43

Data are expressed as the mean ± standard deviation; values in the same row having different letters differ significantly (*p* < 0.05).

Physico-chemical parameters provide important information regarding storage, structural stability and quality of seed oils. The physico-chemical properties of the investigated oils (Table 4) are in agreement with those reported earlier for *L. sativum* [26,27]. The color of an oil is an important feature which often determines the consumers’ acceptability of the product. The oil color observed for *L. sativum* is dirty yellow, while that of *I. hederacea* is light yellow. Oil color is mainly due to the presence of some pigments like chlorophyll and carotenoids which are unintentionally co-extracted during the oil extraction process. The high refractive index values (1.47 ± 0.03 to 1.47 ± 0.08) are an indication of substantial unsaturation in these oils. The refractive index also provides useful information about the purity of oils. Each oil has certain range for this parameter and deviation of the data from the set specification may indicate adulteration of oil [28]. Like other parameters there is limited information on oil physico-chemical characteristics of *I. hederacea* seed oil however our results are comparable to some investigated parameters [29]. Oil composition (Table 5) showed a high content of triacylglycerols, *i.e.*, 80.2 ± 0.51 and 90.00 ± 0.62 g/100g for *I. hederacea* and *L. sativum* respectively. Hydrocarbons and waxes were observed to be present in the lowest amount in both oils. Seed oils can be separated into the various classes on the basis of their relative polarity by using various chromatographic methods, mostly by thin-layer chromatography (TLC). Although this technique is time-consuming, yet it is the most used to study oil classes. Free fatty acids are the products of hydrolysis, formed either due to chemical (due to presence of moisture) or lypolytic hydrolysis (due to presence of natural enzymes, especially lipases). Lower free fatty acid contents (1.20 ± 0.42 to 0.58 ± 0.35 g/100g for *I. hederacea* and *L. sativum*, respectively, indicate a low degree of hydrolysis and thus good oil quality. The low free fatty acid value of both oils also indicates that oils are rich in triglycerides which is a basic and desirable property of edible oils [30]. 

**Table 4:** Physico-chemical parameters of oils of *I. hederacea* and *L. sativum*.

Parameter	*Ipomoea hederacea*	*Lepidium sativum*
Color	Light yellow	Dirty Yellow
Refractive index	1.47 ± 0.03 a	1.47 ± 0.08 a
Specific gravity	0.92 ± 0.04 a	0.82 ± 0.06 a
Unsaponifiable matter	1.73 ± 0.11 a	0.57 ± 0.02 b
Acid value	2.98 ± 0.08 a	1.04 ± 0.05 b
Saponification value	190.48 ± 0.67 a	179.03 ± 0.73 b

Data are expressed as the mean ± standard deviation; values in the same row having different letters differ significantly (*p* < 0.05).

**Table 5:** Composition (%) of oils of *I. hederacea* and *L. sativum*.

Oil class	*Ipomoea hederacea*	*Lepidium sativum*
Hydrocarbons + waxes	0.40 ± 0.23 a	0.30 ± 0.04 a
Steryl esters	0.60 ±0.34 a	0.70 ± 0.07 a
Triacylglycerols	80.20 ± 0.51 b	90.00 ± 0.62 a
Free fatty acid	1.20 ± 0.42 a	0.58 ± 0.35 b
Diglycerols	0.70 ± 0.08 a	0.60 ± 0.58 a
Monoglycerols	1.00 ± 0.37 a	0.10 ± 0.63 b
Polar lipids	6.50 ± 0.91 a	1.70 ± 0.21 b
Phospholipids	4.80 ± 0.82 a	1.40 ± 0.36 b
Unidentified	4.60 ± 0.57 a	4.63 ± 0.67 a

Data are expressed as the mean ± standard deviation; values in the same row having different letters differ significantly (*p* < 0.05).

The fatty acid composition of *I. hederacea* seed oil is in agreement to that reported earlier [31]. Unsaturated fatty acids are found in higher amounts in both oils. Palmitic acid was observed as the most abundant saturated fatty acid, in amounts of 17.03 ± 0.89 and 10.3 ± 0.12 g/100g for *I. hederacea* and *L. sativum*, respectively. Similarly palmitoleic acid was observed as being the least abundant unsaturated fatty acid, with values of 1.10 ± 0.62 and 0.70 ± 0.30 g/100g for *I. hederacea* and *L. sativum* respectively. Fatty acid composition (Table 6) revealed high content of linoleic acid (52%) in *I. hederacea*, while α-linoleic acid (32.18%) and oleic acid (30.5%) were the most abundant fatty acids in the oil of *L. sativum*. Higher intake of oleic acid is associated with decreased risk of coronary heart disease caused by high cholesterol level in blood [32]. The fatty acid composition of the seed oils of these two plant species is interesting from the nutritional point of view for their higher contents of unsaturated fatty acids, especially *L. sativum* rich in ω-3 fatty acids which are is beneficial for health. 

**Table 6:** Fatty acid profile (%) of *I. hederacea* and *L. sativum*.

Fatty acid	*Ipomoea hederacea*	*Lepidium sativum*
Palmitic acid (16:0)	17.03 ± 0.89 a	10.30 ± 0.12 b
Palmitoleic acid (16:1)	1.10 ± 0.62 a	0.70 ± 0.30 b
Stearic acid (18:0)	6.00 ± 0.56 a	1.90 ± 0.19 b
Oleic acid (18:1)	19.50 ± 0.37 b	30.50 ± 0.16 a
Linoleic acid (18:2)	52.09 ± 0.48 a	8.60 ± 0.38 b
α-Linolenic acid (18:3)	4.28 ± 0.92 b	32.18 ± 0.59 a
Arachidic acid (20:0)	-	2.10 ± 0.57
Eicosaenoic acid (20:1)	-	13.40 ± 0.66

Data are expressed as the mean ± standard deviation; values in the same row having different letters differ significantly (*p* < 0.05).

Tocopherols are natural antioxidants that inhibit oil oxidation. Tocopherols act as biological scavengers of free radicals and could prevent diseases, besides possessing an important nutritional function for human beings as a source of vitamin E [33,34]. Total tocopherol contents observed were 33.10 ± 0.56 and 139.73 ± 0.91 mg/100g for *I. hederacea* and *L. sativum*, respectively. γ-Tocopherol was found in the highest amount in seed oil of *I. hederacea*, while δ-tocopherol was the most abundant in the seed oil of *L. sativum* (Table 7). High amounts of tocopherols can be interesting for the stabilization of fats and oils against oxidative deterioration and for applications in dietary, pharmaceutical, or biomedical products. Results presented are in agreement with those reported earlier [35,36,37].

**Table 7:** Tocopherol profile (mg/100g) of oils of *I. hederacea* and *L. sativum*.

Tocopherol	*Ipomoea hederacea*	*Lepidium sativum*
α-Tocopherol	0.50 ± 0.21 b	17.19 ± 0.52 a
β-Tocopherol	1.60 ± 0.21 a	0.10 ± 0.08 b
γ-Tocopherol	28.70 ± 0.14 a	10.88 ± 0.72 b
δ-Tocopherol	2.30 ± 0.34 b	111.56 ± 0.37 a
Total	33.10 ± 0.56 b	139.73 ± 0.91 a

Data are expressed as the mean ± standard deviation; values in the same row having different letters differ significantly (*p* < 0.05).

The samples were subjected to antioxidant capacity screening, using three different testing methods. The extracts of *I. hederacea* and *L. sativum* showed good antioxidant capacity, reducing different types of radicals (Table 8). In general, traditional spectrophotometric assays provide simple and fast screening methods to quantify total phenolic compounds in plant samples. However, due to the complexity of plant phenolics and different reactivity of phenols toward assay reagents, these results should be reinforced by the HPLC analysis, currently the most popular and reliable technique for analysis of phenolic compounds. Using this technique, coupled with mass spectrometer, tentative identification of phenolic compounds based on their mass spectra has been performed (Table 9).

**Table 8:** Antioxidant activity of plant extract of *I. hederacea* and *L. sativum*.

Antioxidant Assay	*Ipomoea hederacea*	*Lepidium sativum*
Total phenol content (mg CAE/g)	14.33 ± 0.09 b	120.26 ± 1.52 a
TEAC (µmol TE/g)	93.57 ± 2.42 b	168.10 ± 1.32 a
FRAP (µmol Fe++/g)	278.24 ± 9.74 b	1317.04 ± 5.74 a
TRAP (µmol TE/g)	69.02 ± 2.92 b	506.40 ± 14.87 a

Data are expressed as the mean ± standard deviation; values in the same row having different letters differ significantly (*p* < 0.05).

**Table 9:** Tentative identification of phenolic compounds based on their mass spectral characteristics present in *I. hederacea* and *L. sativum*.

Compound	[M−H]^−^ ( *m/z*)	Qualifier ions ( *m/z*)	*I. hederacea*	*L. sativum*
Gallic acid	169	125	-	+
Ferulic acid	193	134	+	-
Protocatechuic acid	153	109	-	+
Coumaric acid	163	119	-	+
Caffeic acid	179	135	-	+
Coumaric acid-hexoside	325	163, 119	+	+
Caffeic acid-hexoside	341	179, 135	-	+
Ferulic acid-hexoside	355	193, 134	+	+
Sinapic acid-hexoside	385	223,149	+	-
Vanillic acid-hexoside	329	167	-	+
Caffeoylquinic acid	353	191, 179, 173, 135	-	+
Coumaroylquinic acid	337	191, 173	-	+
Kaemferol-hexoside	447	285	-	+
Quercetin-hexoside	463	301	-	+
Kaemferol-glucuronide	461	285	-	+
Quercetin	301	151	-	+

**Figure 1:**
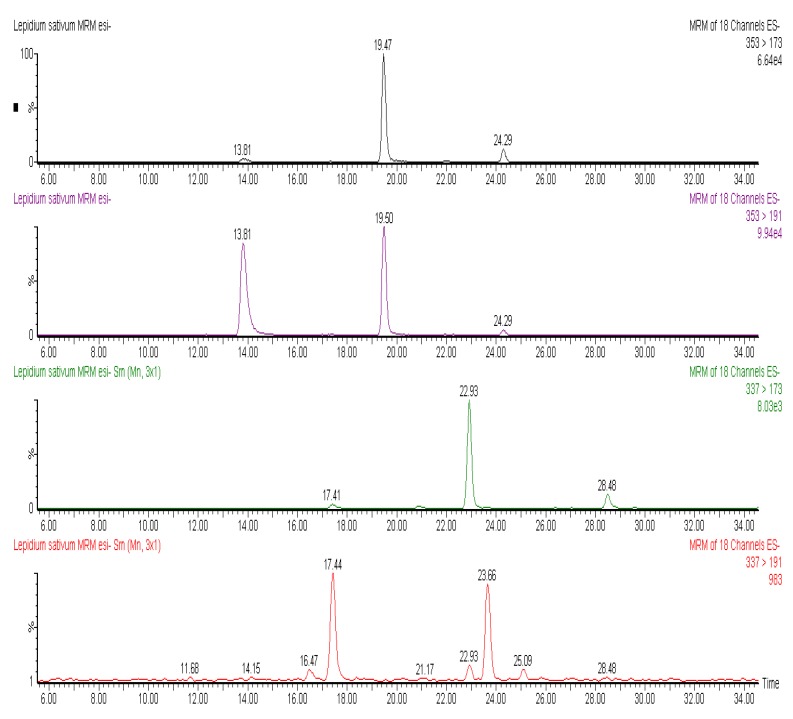
MRM chromatograms of caffeoylquinic acid. It is possible to note at least three isomers (at 13.81, 19.47 and 24.29 min), identified through the loss and ionization quinic acid (353 > 191) and (353 > 173) derived from loss of H_2_O from formed quinic acid. MRM chromatograms of coumaroylquinic acid, identified through the loss and ionization quinic acid (337 > 191) and (337 > 173) derived from loss of H_2_O from formed quinic acid.

**Figure 2:**
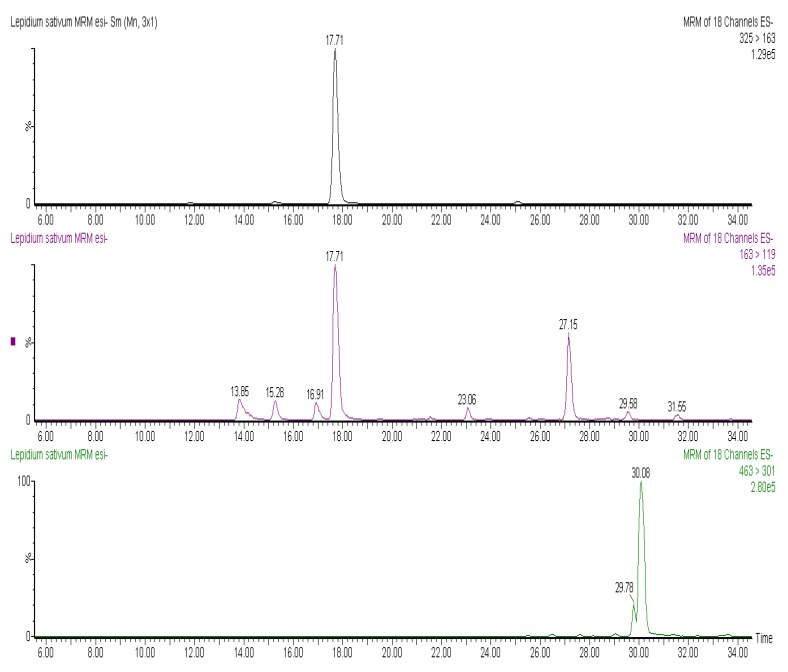
MRM chromatograms of coumaric acid-hexosides. It is possible to note at least two isomers (at 15.28 and 17.71 min), identified through the loss of hexose moiety (325 > 163) and further fragmentation of coumaric acid (163 > 119). MRM chromatogram of quercetin-hexosides (463 > 301), identified through the loss of sugar moiety and resultant ionization of quercetin.

More phenolics have been identified in *L. sativum* than *I. hederacea*. In Figure 1, the chromatographic profile of some polyphenols identified in *L. sativum* is reported. At least three isomers of caffeoylquinic acid were identified in the seeds of *L. sativum*. These isomers had a negatively charged molecular ion ([M−H]^−^) at *m/z* 353 and MS2 ions deriving from loss of the caffeoyl moiety and resulting ionization of free quinic acid (*m/z* 191) and quinic acid with a loss of water molecule (*m/z* 173). Among chlorogenic acids, coumaroylquinic acids showed a [M−H]^−^ at *m/z* 337, and were characterized by the same fragmentation patterns of caffeoylquinic acids, involving the breakdown of hydroxycinnamate-quinic acid linkages. Moreover, coumaric acid was recovered in esterified form with hexose groups, with a [M−H]^−^ at *m/z* 325, that generated by tandem mass fragmentation the free coumaric acid after loss of a hexose moiety (*m/z* 163) and further fragmentation of free hydroxycinnamic acid (*m/z* 119) (Figure 2). 

Besides phenolic acids, *L. sativum* contained several flavonoids, including two quercetin-hexosides that shared [M−H]^−^ at *m/z* 463, identified through the loss of sugar moieties (probably glucose and galactose units) and resultant ionization of quercetin at *m/z* 301, as reported in Figure 2. As for the phenolic profile of *I. hederacea*, only free ferulic acid and sinapic acid-hexosides were identified in this plant, while ferulic and coumaric acids were presented in esterified form with hexose groups, like in *L. sativum*. 

Total antioxidant capacity, measured by three assays, and total phenolic content, measured using the Folin–Ciocalteu colorimetric method, of analyzed plant extracts are reported in Table 8. *L. sativum* seeds had a higher content of TPC (120.26 mg CAE/g) than *Ipomea hederacea* seeds and this is consistent with the higher number of phenolics identified in these seeds (Table 9). Few studies have evaluated the content of TPC in these plants. Compared to our results [38], analyzing the hydroalcoholic extracts of edible plants from Calabria (Italy), we found a lower content of TPC in *L. sativum* leaves, whereas Aziz-ur-Rehman *et al*. found a higher TPC value in *I. hederacea* extracted with different solvents and collected from the Kotli region in Pakistan [39]. Due to the higher content of TPC and total tocopherols, *L. sativum* showed higher values of total antioxidant capacity (TAC), regardless of the applied assay (Table 8), with respect to *I. hederacea*. TEAC and FRAP values of the analyzed *L. sativum* were high when compared to those found in Indian *L. sativum* seeds, which, accordingly, showed a lower content of TPC than that found in our sample [40]. Similarly, FRAP values of *I. hederacea* were higher than those reported by Aziz-ur-Rehman *et al.* [39]. It is well known that the antioxidant capacity and phenolic content of plants depend on several factors such as different genotype, growing condition, agronomic practices employed, season, maturity, post-harvest storage and processing conditions and solvent used for extraction*.* These factors may explain the differences found among our samples and those analyzed in previous studies.

## 3. Experimental

### 3.1. Plant Material

*Ipomoea hederacea* and *Lepidium sativum* seeds were procured from the Department of Agronomy, Bahauddin Zakariya University, Multan, Pakistan. Seeds of both plants were stored in stainless-steel containers at 4 °C prior to analysis. 

### 3.2. Proximate Analysis

Moisture, lipids, ash, protein, fibre and carbohydrates were determined according to the AOAC methods [41]. 

### 3.3. Mineral Analysis

The seeds were incinerated at 450 °C for 12 h in a muffle furnace and an acid digestate was prepared by oxidizing each sub-sample with a nitric/perchloric acid (2:1) mixture. Aliquots were used to estimate Na and K by flame photometry (Flame Photometer Model-EEL). The minerals, such as calcium, manganese, magnesium, zinc, iron and copper, were determined with an atomic absorption spectrophotometer (Perkin–Elmer Model 5000) while phosphorus was determined by the phosphovanado-molybdate (yellow) method [42]. The samples were quantified against standard solutions of known concentration that were analyzed concurrently [43].

### 3.4. Amino Acid Analysis

Samples (300 mg) were hydrolyzed with 6 M HCl in an evacuated test tube for 24 h at 105 °C. The dried residue was dissolved in citrate buffer (pH 2.2) after flash evaporation. Aliquots were analyzed in an automatic amino acid analyzer (Hitachi Perkin–Elmer Model KLA 3B), using the buffer system described earlier [44]. Methionine and cystine were analysed separately after performic acid treatment and subsequent hydrolysis with HCl. Tryptophan was determined after alkali (NaOH) hydrolysis by the colorimetric method [45].

### 3.5. Extraction of Oil and Physical Properties

The seeds were ground to flour and were passed through a 60-mesh sieve. The seed powder was extracted with a mixture of *n*-hexane/2-propanol (3:1, V/V) in a Soxhlet apparatus for 6 h [46]. The color of the oil was noted, at room temperature, by visual inspection. Determination of refractive index (RI), acid value, saponification value (SV) and unsaponifiable matter (UM) of the extracted oil was carried out by standard IUPAC methods for the analysis of oils [47].

### 3.6. Oil Classes (%)

Oil samples were fractionated by a reported method [48] using silica gel, G 60 Merck type 5721 and 20 cm × 20 cm glass plates with 0.25 mm thickness. The developing solvent system was *n*-hexane/ diethyl ether/ glacial acetic acid (80:20:2, V/V/V). The separated oil fractions were visualized by exposure to iodine vapor in a closed chamber after drying. All fractions were identified on thin layer plates by comparing their Rf values with those of known standards. For quantitative analysis, the different fractions were scanned by using Shimadzu TLC-Scanner (C-S-910). The area under each peak was measured by the triangulation method [33]. The percentage of each component was calculated with regard to the total area by a reported procedure [49] as follows:



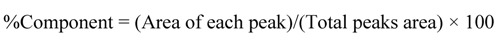



### 3.7. Fatty Acid (FA) Composition

Fatty acid methyl esters (FAMEs) were prepared according to the standard IUPAC method 2.301 [46] and analyzed on a Shimadzu 17-A gas chromatograph equipped with a flame ionization detector (FID). Separation was done on a SP 2330 capillary column (30 m × 0.32 mm × 0.25 μm; Supelco, Bellefonte, PA, USA). Nitrogen was used as a carrier gas at a flow rate of 3.0 mL/min. Column temperature was programmed from 180 to 220 °C at the rate of 3 °C/min. Initial and final temperatures were held for 2 and 10 min, respectively. Injector and detector were kept at 230 and 250 °C, respectively. A sample volume of 1.0 μL was injected with the split ratio of 1:75. FAMEs were identified by comparing their relative and absolute retention times to those of authentic standards. The quantification was done by a Chromatography Station for Windows (CSW32) data handling software (Data Apex Ltd. CZ-158 00 Prague, Czech Republic). The fatty acid composition was reported as a relative percentage of the total peak area.

### 3.8. Tocopherol Contents

For determination of tocopherols, 250 mg of oil (obtained as mentioned in Section 3.5) was dissolved in *n*-heptane (25 mL) and used for the HPLC analysis which was conducted using a Merck-Hitachi low-pressure gradient system, fitted with an L-6000 pump, a Merck-Hitachi F-1000 Fluorescence Spectrophotometer (detector wavelengths for excitation 295 nm, for emission 330 nm) and a D-2500 integration system; samples (20 μL) were injected by a Merck 655-A40 Autosampler onto a diol phase HPLC column 25 cm × 4.6 mm ID (Merck, Darmstadt, Germany) using a flow rate of 1.3 mL/min. The mobile phase used was *n*-heptane/*tert*-butyl methyl ether (99 + 1, v/v) [50]. 

### 3.9. Sample Preparation for Antioxidant Assays

The seeds were ground to flour with an IKA® All Basic mill (IKA Works Inc., Wilmington, NC, USA) and passed through a 60-mesh sieve. The flour was macerated with aqueous methanolic mixture (3 Litre, 80:20; v/v), at room temperature for fifteen days with occasional shaking. The process was repeated three times with same quantity of solvent mixture. The extracts obtained were combined, filtered through filter paper under vacuum and concentrated under reduced pressure on a rotary evaporator (model Q-344B-Quimis, Sao Paulo, Brazil) using a warm water bath (model Q-214M2-Quimis) to obtain a thick gummy mass, which was further dried in a dessicator and stored in air-tight vial till further use .

#### 3.9.1. Total Phenolic Content

A weighed amount of extract sample (between 50 and 130 mg depending on the sample) was dissolved with acidified methanol (10 mL, 1% formic acid). The extracts were kept at −20 °C at dark prior to the analysis. The content of phenolic compounds was determined using the Folin-Ciocalteu method; based on the reduction of phosphotungstate-phosphomolybdate complex by phenolics to a blue reaction product that absorbance was measured at 760 nm [51]. The data were calculated according to standard curve of catechin (0.01–0.20 mg/mL), and were expressed as mg of catechin equivalents (CE) per gram of extract. 

#### 3.9.2. HPLC-ESI-MS/MS Analysis of Phenolic Compounds

Phenolic compounds were analysed using a Water 2695 Alliance separation module equipped with a Micromass Quattro Micro API mass spectrometer fitted with an electrospray interface (ESI) (Waters, Milford, MA, USA). A preliminary investigation on phenolic profiles of selected plants was carried out by means of MS scan analysis, operating in negative ion mode from 100 to 1000 mass-to-charge ratio (*m/z*). Then, different Multiple Reaction Monitoring (MRM) methods were developed for all sample types, based on the obtained MS scan data. Separations were performed using a Waters Atlantis dC18 3 µm (2.1 × 150 mm) reverse phase column (Waters), with the mobile phase, pumped at a flow rate of 0.17 mL/min. The mobile phase was a 30-min linear gradient of 5 to 30% acetonitrile in 1% aqueous formic acid. The ESI source worked in negative ionisation mode. Source temperature was 120 °C, desolvation temperature was 350 °C, capillary voltage was 2.8 kV, cone voltage was 35 V, desolvation gas (N2) 750 L/h, cone gas (N2) 50 L/h. The collision energy for MS/MS identifications was set at 30 eV, and the collision gas used was argon.

#### 3.9.3. Total Antioxidant Capacity

Plant extracts were analysed for their antioxidant capacity by three different assays described before: 2,2′-azino-bis(3-ethylbenzthiazoline-6-sulphonic acid) radical-scavenging activity (ABTS method), [52], ferric reducing antioxidant power (FRAP) assay, [53], and total radical-trapping antioxidant parameter (TRAP) assay [54]. The TEAC and TRAP values were expressed as micromoles of Trolox per g of plant extract, FRAP values were expressed as micromoles of Fe^2+^ equivalents per g of plant extract.

### 3.10. Statistical Analysis

All analyses were performed in triplicate and values expressed as the mean ± standard deviation. Data analysis was carried out using the analysis of variance and MSTATC statistical computer package [55].

## 4. Conclusions

The content of biologically active compounds, as well as the antioxidant capacity of *I. hederacea* and *L. sativum* have been investigated in this study. Our findings indicate that seeds of both plants are a good source of amino acids, minerals, fatty acids and have the ability to act *in vitro* as antioxidants. This may be due to their high content of phenolic compounds. These plants may warrant further investigation for their potential preventive effects towards chronic diseases and should be further investigated as interesting ingredients for new functional food formulations.
